# M protein typing of Thai group A streptococcal isolates by PCR-Restriction fragment length polymorphism analysis

**DOI:** 10.1186/1471-2180-5-63

**Published:** 2005-10-16

**Authors:** Nonglak Yoonim, Colleen Olive, Chulabhorn Pruksachatkunakorn, Michael F Good, Sumalee Pruksakorn

**Affiliations:** 1 Department of Microbiology, Faculty of Medicine, Chiang Mai University, Chiang Mai, Thailand; 2 Department of Pediatrics, Faculty of Medicine, Chiang Mai University, Chiang Mai, Thailand; 3 Queensland Institute of Medical Research, 300 Herston Road, Herston, Brisbane, QLD, Australia

## Abstract

**Background:**

Group A streptococcal (GAS) infections can lead to the development of severe post-infectious sequelae, such as rheumatic fever (RF) and rheumatic heart disease (RHD). RF and RHD are a major health concern in developing countries, and in indigenous populations of developed nations. The majority of GAS isolates are M protein-nontypeable (MNT) by standard serotyping. However, GAS typing is a necessary tool in the epidemiologically analysis of GAS and provides useful information for vaccine development. Although DNA sequencing is the most conclusive method for M protein typing, this is not a feasible approach especially in developing countries. To overcome this problem, we have developed a polymerase chain reaction-restriction fragment length polymorphism (PCR-RFLP)-based assay for molecular typing the M protein gene (*emm*) of GAS.

**Results:**

Using one pair of primers, 13 known GAS M types showed one to four bands of PCR products and after digestion with *Alu *I, they gave different RFLP patterns. Of 106 GAS isolates examined from the normal Thai population and from patients with GAS-associated complications including RHD, 95 isolates gave RFLP patterns that corresponded to the 13 known M types. Only 11 isolates gave RFLP patterns that differed from the 13 known M types. These were then analyzed by DNA sequencing and six additional M types were identified. In addition, we found that M93 GAS was the most common M type in the population studied, and is consistent with a previous study of Thai GAS isolates.

**Conclusion:**

PCR-RFLP analysis has the potential for the rapid screening of different GAS M types and is therefore considerably advantageous as an alternative M typing approach in developing countries in which GAS is endemic.

## Background

*Streptococcus pyogenes *or group A streptococcus (GAS) causes a number of clinical manifestations and diseases including sore throat, pyoderma, necrotizing fasciitis, toxic-shock syndrome, and the post-infectious sequelae – rheumatic fever (RF) and rheumatic heart disease (RHD) [1]. RF and RHD are a major health concern worldwide but especially in indigenous communities within developed countries, and in populations of developing countries [2]. The GAS surface M protein is known to prevent opsonophagocytosis and is a major virulence factor in GAS infection [1]. The N-terminal region of the M protein is highly variable between different GAS strains and contains a type-specific moiety, the antigenic variation of which forms the basis for the classical M protein serological typing of GAS. However, there are disadvantages with this method of typing including ambiguities in the results, the emergence of new M types, and the high rates of M protein-nontypeable (MNT) strains [3] largely due to the unavailability of specific typing antisera. As a consequence, there has been a surge of interest in the development of alternative methods for M typing utilizing molecular technologies.

Several methods have been developed for GAS typing such as enzyme electrophoretic polymorphism [4-6], genomic typing methods such as RAPD [7], 16s rDNA typing [8], RFLP analysis [9-12], vir typing [13,14], DNA hybridization using N-terminal sequences of the M protein gene (*emm*) as oligonucleotide probes [15,16], polymerase chain reaction (PCR)-enzyme linked immunosorbant assay [17], and PCR M typing using type-specific oligonucleotide primers for PCR amplification of the N-terminal region of the *emm *gene [18]. PCR-RFLP analysis which utilizes PCR to amplify the *emm *gene amplicons encoding the M protein prior to digestion with restriction endonucleases has been used for specific molecular M typing methods [19-22], as well as multilocus sequence typing (MLST) [23]. N-terminal sequencing of the M protein gene, however, is the most conclusive method for typing of GAS [24,25], This method of typing is not an option in most laboratories in developing countries worldwide, due to limited resources, and therefore an alternative approach is required for the identification of GAS types. The purpose of this study was therefore to analyze the N-terminal regions of the *emm *gene of Thai GAS isolates using PCR-RFLP analysis. PCR products were digested with an appropriate restriction enzyme and the fragments analyzed by polyacrylamide gel electrophoresis (PAGE).

## Results and discussion

The N- and C-terminal region of the *emm *gene was amplified by PCR from all of the 119 GAS isolates. The amplicons consisted of one to four bands which varied from approximately 450 to 1200 bp depending on the M types and GAS strains (Fig. 2A). However, some M types had similar sizes of their *emm *amplicons, but gave distinct bands after digestion with the restriction enzyme, *Alu *I. This revealed RFLP patterns consisting of two to nine distinct fragments, which ranged from approximately 25 to 700 bp depending on the variability of DNA sequences in the *emm *amplicons of different M types (Fig. 2B). The RFLP patterns of 95 unknown GAS M types corresponded to 13 known M types (Table 1, Fig. 2B). The M types of all isolates represented by the PCR-RFLP patterns were confirmed by DNA sequencing analysis. All isolates were sequenced independently of the PCR-RFLP analysis which was carried out blinded. The two sets of data were then compared. Only 11 isolates gave patterns that did not corresponded to the 13 known M types. The isolates that could not be identified by PCR-RFLP analysis were also analyzed by DNA sequencing. Six M types that differed from the 13 known M types were obtained from the DNA sequencing analysis (M4, M33, *emm*58.5, M102, M109 and M89). Although these M protein DNA sequences were ≥95% similar to the data in GenBank, we found both point mutations and deletions in these isolates. M89 has a single-base substitution (T→C) that resulted in a new *Alu *I restriction site whereas M4 and M102 have 21-base and 33-base deletions, respectively. Alignment of the amino acid sequences of M4 and M102 with reference strains in GenBank, demonstrated ≥95% similarity (Fig. 3).

For decades serotyping has been the method of choice for GAS M typing. However, serotyping is time consuming and it is often difficult to produce high-titer M type-specific antisera, and therefore this technique is limited to a few laboratories in the world. To overcome these limitations, genomic typing methods have been developed [7-12], as well as specific M typing methods [15-18]. RFLP analysis of the *emm *gene revealed different RFLP patterns among GAS isolates with the same M type [19-22]. Therefore, it could potentially be used to differentiate among isolates with the same M type, but which are not clonal, and may originate from different geographical locations or populations.

In this study, we applied PCR-RFLP analysis for GAS M typing. PCR products derived from the amplification of the N- and C-terminal region of the *emm *gene and digested with the restriction enzyme, *Alu *I, produced different RFLP patterns among different M types. Ninety five clinical isolates could be compared with known M types by their RFLP patterns. Only 11 isolates had novel RFLP patterns and required sequencing to confirm the M types. Our results showed that M93 GAS was the most common GAS strain isolated from the population studied with 22 isolates (20.75%) having this type, and is consistent with a previous study of Thai GAS isolates [3]. Other M types that represented more than 10% of the Thai GAS isolates were M1 and M44/61, whereas M66, *emm*58.5, M89 and M102 were quite rare (0.94%). Furthermore, the specificity of the PCR-RFLP analysis was confirmed by sequencing of the N-terminal region of the *emm *gene. The majority of isolates were sequenced for each M type. M4, M33, M48, *emm*58.5, M66, M81, M89, M102, M109, M112 and st11014 were sequenced in all isolates. M1, M11, M12, M25, M44, M49, M93 and M104 were sequenced in 8 of 16, 7 of 8, 2 of 3, 3 of 10, 3 of 11, 1 of 2, 11 of 22 and 2 of 3 isolates, respectively. All isolates were sequenced independently of the PCR-RFLP analysis which was carried out blinded. The two sets of data were then compared. The results obtained from the PCR-RFLP analysis were in agreement with the sequencing data.

In comparison with other genotyping methods based on RFLP analysis [8-10,19-22], the PCR-RFLP analysis used here has some technical advantages. Only one pair of PCR primers is required for the amplification of all of the GAS isolates, and the digested PCR products can be discriminated using standard PAGE which is easy to perform, interpret and is less time consuming. In addition, compared with sequencing analysis [24,25], the PCR-RFLP analysis is technically less demanding and more economical. Therefore, with this simple and rapid protocol, GAS typing can be used in any laboratory in which PCR is routinely used, and is particularly useful for typing large numbers of GAS isolates. However, our findings are based on a relatively small number of *emm *types isolated in a relatively limited geographic region over a relatively short period of time. Greater geographic and temporal diversity may result in greater clonal diversity thus complicating interpretation of RFLP patterns. Therefore, we suggest that each laboratory should make their own reference RFLP patterns from M types that circulate in their region. However, there are several problems that may occur such as point mutation and deletion. These result in the strains that can not be identified. In this case, it should then be typed by sequence analysis and added these variants into their reference RFLP patterns. In addition, to confirm validation of their results, sequencing a subset of their analyzed isolated should be done periodically.

## Conclusion

PCR-RFLP analysis is a rapid, economical and practical genotyping method for determining the M type of GAS. It is therefore particularly suited as a method of choice in developing countries in which GAS is endemic and the majority of GAS isolates are M protein-nontypeable by conventional serological typing.

## Methods

### Bacteria

One hundred and six strains of GAS isolated from the normal Thai population, patients with sore throat, RHD or impetigo from 1985, 1990, 1995, 2000, 2003 and 2004, and 13 known GAS M serotypes, were included in the study.

### DNA isolation and PCR

**Figure 1:**
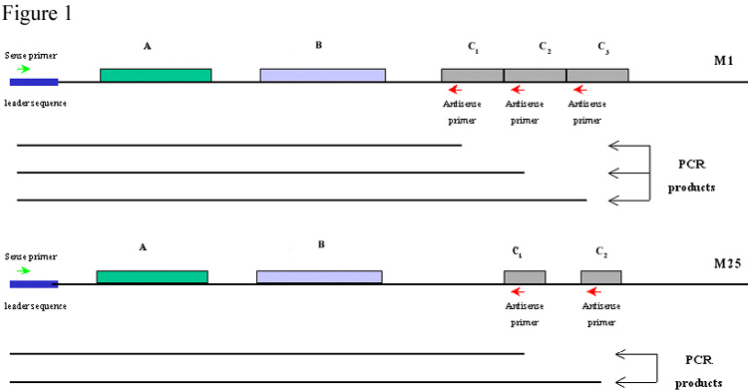
Diagrammatic representation of the *emm*1 and *emm*25 genes of M1 and M25 proteins, respectively, showing the different A-, B- and C-repeat regions encoding the M protein. The location of the sense and antisense primers used for PCR amplification of the *emm *gene are indicated as well as the different *emm *gene amplicons (PCR products) that arise depending upon the number of C-repeat regions within the *emm *gene.

**Figure 2:**
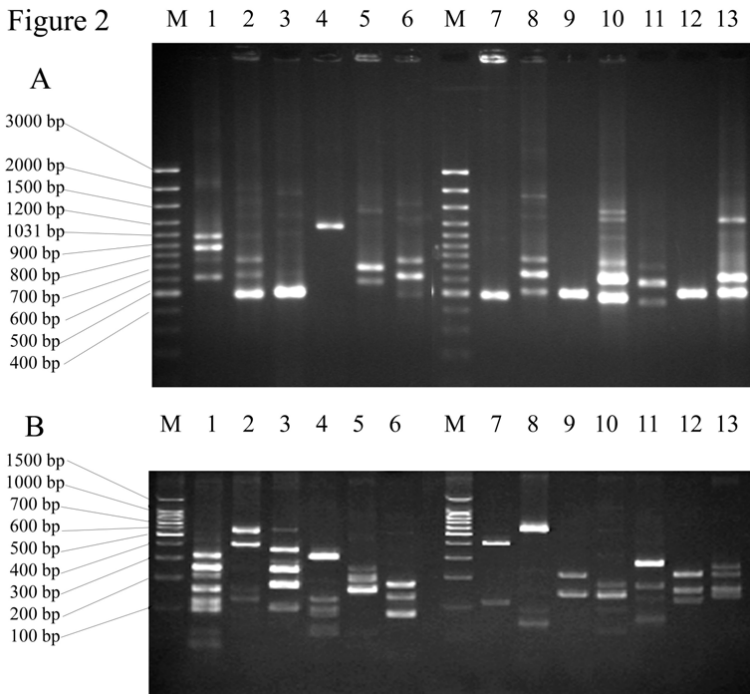
Agarose gel electrophoresis of *emm *gene amplicon PCR products (A), and polyacrylamide gel electrophoresis RFLP patterns using *Alu *I restriction enzyme (B). The size differences of PCR products and RFLP patterns of M1, M11, M49, M12, M25, M48, M112, st11014, M44/61, M93, M81, M104 and M66 are shown in lanes 1–13, respectively. Lane M represents a 100 bp ladder.

**Figure 3:**
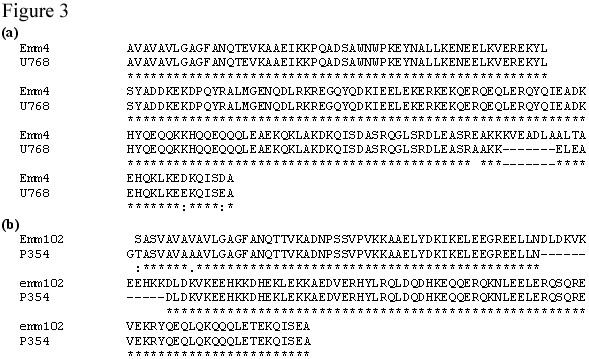
Alignment of amino acid sequences from U768 with M4 (*emm*4 gene) and P354 with M102 (*emm*102 gene) in GenBank.

DNA was isolated from GAS based on the method previously described [3]. The sense primer (CAGTATTCGCTTAGAAAATTAAAA) was derived from the conserved leader sequence of *emm *gene [26]. The antisense primer (CCCTTACGGCTTGC TTCTGA) was derived from the C-repeat region of the *emm *gene [3]. The PCR conditions were as follows: denaturation at 94°C for 30 s, annealing at 45°C for 30 s, and extension at 72°C for 2 min for 35 cycles. These primers were also used for sequencing analysis. Using these primers, up to four *emm *gene amplicons can be generated for a particular GAS strain following PCR amplification depending on the number of C-repeat regions in the M protein (Fig. 1).

### PCR-RFLP analysis

PCR-RFLP analysis can be used to type different *emm *genes. PCR amplification and digestion with appropriate restriction enzymes is expected to give different RFLP patterns among GAS. Based on the *emm *gene sequences in the GenBank database and analysis using the restriction mapper program [27], we chose the restriction enzyme, *Alu *I, because almost all sequences contained at least one *Alu *I site in different positions in their sequences. Therefore, digestion with *Alu *I is likely to give different RFLP patterns among GAS M types. Following PCR, the products (*emm *amplicons) were analyzed by electrophoresis on a 1% agarose gel in TBE buffer. The sizes of PCR products were compared with the 100 bp standard marker and recorded following staining with ethidium bromide. PCR products were then partially purified by ethanol precipitation prior to quantitation and digestion with the *Alu *I restriction enzyme according to the manufacturer's specifications. Digested PCR products were separated on a 15% polyacrylamide gel in TBE buffer and compared with the 100 bp standard marker following staining with ethidium bromide. A log-linear standard curve was initially generated in the Excel program using the known marker sizes versus gel running distance. The equation from the standard curve was then used to estimate the sizes of experimental bands.

### DNA sequencing analysis

PCR products were sequenced using the ABI Dye Terminator Cycle Sequencing Ready Reaction Kit according to the manufacturer's instructions (The Perkin-Elmer Corporation) and analyzed using an ABI 310 automated sequencer (The Perkin-Elmer Corporation). DNA sequences were transferred to the DNASIS program for sequence comparison between isolates. The BLAST 2 program (NCBI) was used to determine the levels of DNA homology with published sequences in the GenBank database.

## Authors' contributions

NY carried out the microbiologic experiments, performed the molecular genetic analysis, participated in the sequence alignment, interpretation of data and drafted the manuscript. CO contributed to the editing of the manuscript. CP provided clinical specimens and clinical support. MFG provided PCR primers and molecular reagents. SP conceived of the study, and participated in its design and coordination. All authors read and approved the final manuscript.

## Figures and Tables

**Table 1:** PCR products and *Alu *I restriction fragment sizes and distribution in the Thai GAS isolates studied.

M type	Size of PCR product (bp)	Size of restriction fragment (bp)	No. (%) of isolates from PCR-RFLP analysis	M type	Size of PCR product (bp)	Size of restriction fragment (bp)	No. (%) of isolates from PCR-RFLP analysis
M1	1071	354	16 (15.09%)	M49	450	336	2 (1.89%)
	892	257				252	
	744	218				196	
	592	147				135	
		110				89	
		87		*Emm*58.5	545	370	1 (0.94%)
		47				185	
		37		M66	566	252	1 (0.94%)
		29			450	206	
M4	747	314	2 (1.89%)			148	
	609	174				123	
	476	57		M81	649	322	7 (6.60%)
		47			493	185	
M11	710	593	8 (7.55%)		375	127	
	592	434				84	
	450	147				62	
		117		M89	404	350	1 (0.94%)
M12	1121	349	3 (2.83%)			77	
		116		M93	679	254	22 (20.75%)
		93			540	227	
		78			411	168	
		59				130	
		51				93	
M25	649	247	10 (9.43%)			53	
	540	203		M102	420	332	1 (0.94%)
		148				92	
		51		M104	450	210	3 (2.83%)
		37				146	
M33	812	347	3 (2.83%)			118	
	670	168		M109	509	386	3 (2.83%)
	541	94				168	
		74		M112	450	435	5 (4.72%)
M44/61	450	212	11 (10.38%)			104	
		133		st11014	744	596	5 (4.72%)
M48	710	162	2 (1.89%)		620	87	
	592	121			493	29	
	471	81					
